# Health related quality of life in an at‐risk population during an intervention study and extended follow‐up ‐ the Finnish Geriatric Intervention Study to Prevent Cognitive Impairment and Disability (FINGER)

**DOI:** 10.1002/alz.090679

**Published:** 2025-01-09

**Authors:** Jenni Lehtisalo, Tiia Ngandu, Francesca Mangialasche, Tiina Laatikainen, Timo Strandberg, Riitta Antikainen, Jaakko Tuomilehto, Hilkka Soininen, Dharma Singh Khalsa, Miia Kivipelto

**Affiliations:** ^1^ University of Eastern Finland, Kuopio Finland; ^2^ Finnish Institute for Health and Welfare, Helsinki Finland; ^3^ Karolinska Institutet, Stockholm Sweden; ^4^ Population Health Unit, Finnish Institute for Health and Welfare, Helsinki Finland; ^5^ Institute of Public Health and Clinical Nutrition, University of Eastern Finland, Kuopio Finland; ^6^ Population Health Unit Finnish Institute for Health and Welfare, Helsinki Finland; ^7^ University of Oulu, Oulu Finland; ^8^ University of Helsinki, Helsinki University Central Hospital, Helsinki Finland; ^9^ Department of Public Health, University of Helsinki, Helsinki Finland; ^10^ Diabetes Research Unit, King Abdulaziz University, Jeddah Saudi Arabia; ^11^ Population Health Unit, Finnish Institute for Health and Welfare, Helsinki Finland; ^12^ South Ostrobothnia Central Hospital, Seinäjoki Finland; ^13^ Alzheimer’s Research and Prevention Foundation, Tucson, AZ USA; ^14^ Imperial College London, London United Kingdom; ^15^ Institute of Public Health, University of Eastern Finland, Kuopio Finland

## Abstract

**Background:**

The multidomain lifestyle intervention in the Finnish Geriatric Intervention Study to Prevent Cognitive Impairment and Disability (FINGER) showed positive effects on health‐related quality of life (HRQL) during the 2‐year intervention, particularly physical functioning. Our aim was to study how these benefits were maintained over an extended follow‐up.

**Method:**

A total of 1259 older adults aged 60‐77 were randomized into multidomain intervention (n = 631) or control groups (n = 628). A validated HRQL scale RAND‐36 with 8 subscales was collected during the original 2‐year intervention (0,1,2 yrs) and during the 11‐year extended follow‐up (7,11 yrs). Emotional well‐being subscale was also available from a mail survey during the COVID19 pandemic (approx. 9 yrs). Changes over time and intervention effect on HRQL were analyzed using mixed‐effects regression.

**Result:**

Altogether 1212 (96%) provided HRQL data at baseline, 1069 (85%) after the intervention, and 501 after 11 years (40%). During the follow‐up mean RAND total score decreased from 76.4 to 69.3 All subscales were below baseline after 7 years, and physical functioning subscales started to decline already after 2 years (physical functioning, role limitations due to physical health, and bodily pain). Intervention related improvement in general health perception at the 1^st^ year was maintained up to 11 years (p‐value for difference in change between groups 0.002), but improvement in physical functioning subscales vanished over time. Emotional well‐being started to decline at the 7‐year visit, without group differences. It temporarily improved during the pandemic (p‐value for change from the 7‐year visit 0.001) but declined back at the 11‐year visit.

**Conclusion:**

Overall HRQL declined over time, as expected with aging. A 2‐year multidomain lifestyle intervention among at‐risk older adults resulted in sustained difference in general health perception, which is an important marker of general health. Lesser decline in physical functioning observed during the intervention was not sustained during the extended follow‐up. In the whole group, emotional well‐being declined later than physical functioning. Temporary improvement during the pandemic could be explained by the timing of the questionnaire (right after lifting restrictions), but it indicates that rapid changes due to external factors have an impact on emotional well‐being of older people.

